# Pulmonary Delivery of Hydroxychloroquine Nanostructured Lipid Carrier as a Potential Treatment of COVID-19

**DOI:** 10.3390/polym14132616

**Published:** 2022-06-28

**Authors:** Ahmed Shaker Ali, Mohsen Geza Alrashedi, Osama Abdelhakim Aly Ahmed, Ibrahim M. Ibrahim

**Affiliations:** 1 Department of Pharmacology, Faculty of Medicine, King Abdulaziz University, Jeddah 21589, Saudi Arabia; profahmedali@gmail.com (A.S.A.); abu_geza@hotmail.com (M.G.A.); 2 Department of Pharmaceutics, Faculty of Pharmacy, Assiut University, Assiut 71515, Egypt; 3 Ministry of Health, Riyadh 12628, Saudi Arabia; 4 Department of Pharmaceutics, Faculty of Pharmacy, King Abdulaziz University, Jeddah 21589, Saudi Arabia; oaahmed@kau.edu.sa

**Keywords:** antiviral, drug delivery, nanoformulations, acute lung injury, bleomycin, pro-inflammatory cytokines

## Abstract

Coronavirus Disease 2019 (COVID-19) is a pandemic caused by severe acute respiratory syndrome coronavirus 2. Pneumonia is considered the most severe and long-term complication of COVID-19. Among other drugs, hydroxychloroquine (HCQ) was repurposed for the management of COVID-19; however, low efficacy and cardiac toxicity of the conventional dosage form limited its use in COVID-19. Therefore, utilizing nanotechnology, a pulmonary delivery system of HCQ was investigated to overcome these limitations. HCQ was formulated in nanostructured lipid carriers (HCQ-NLCs) using the hot emulsification–ultrasonication method. Furthermore, the prepared formulation was evaluated in vitro. Moreover, the efficacy was tested in vivo in a bleomycin-induced acute lung injury mice model. Intriguingly, nanoformulations were given by the intratracheal route for 6 days. HCQ-NLCs showed a mean particle size of 277 nm and a good drug release profile. Remarkably, acute lung injury induced by bleomycin was associated with a marked elevation of inflammatory markers and histological alterations in lung tissues. Astoundingly, all these changes were significantly attenuated with HCQ-NLCs. The pulmonary delivery of HCQ-NLCs likely provided adequate targeting to lung tissues. Nevertheless, there is hope that this novel strategy will eventually lead to the improved effectiveness and diminished probability of alarming adverse drug reactions.

## 1. Introduction

Severe acute respiratory syndrome coronavirus 2 (SARS-CoV-2) is the causative organism of the Coronavirus Disease 2019 (COVID-19) pandemic. It is a highly contagious respiratory disease that has led to at least 6 million deaths and its negative economic impacts. Now, severe COVID-19 is described as complicated viral pneumonia associated with systemic inflammation with multiple organ involvement including the blood vessels, lungs, heart, central nervous system, and other organs [1]. Health authorities around the world have repurposed several drugs to be used in the management of this pandemic. These medications include hydroxychloroquine (HCQ)/chloroquine (CQ), azithromycin, remdesivir, favipiravir, lopinavir/ritonavir, ribavirin, and umifenovir. Most of the repurposed antiviral drugs were found to have a negative risk–benefit balance [2,3]. HCQ is a derivative of 4-aminoquinoline as with chloroquine (CQ). It is an inexpensive drug commonly indicated for the management of malaria and some immune disorders including rheumatoid arthritis and lupus [4,5].

Typically, it is administered by mouth as HCQ sulfate. In the early stages of the deadly pandemic, attention and debates were drawn to this medication owing to its promising activity in the management of COVID-19 [5]. It is believed that HCQ has several modes of action, such as the inhibition of viral entry, as well as the liberation of viral RNA into the host cells, in addition to modulation of the immune system [6,7]. However, most studies indicated its limited efficacy [8]. It was suggested that pulmonary acidosis associated with severe SARS-CoV-2 infection diminishes the access of this weakly basic drug to lung tissues. More limitations of HCQ use in severe COVID-19 patients include the prolonging of the QT interval [6], suppression of the immune system [9], and extreme variability in serum HCQ concentrations [10,11].

Hence, it is crucial to establish an effective approach to overcome these limitations to optimize its use in the management of COVID-19. Alrashedi et al. (2021) suggested that taking anti-infective drugs by inhalation likely increases their effectiveness in the treatment of COVID-19 and reduces their side-effects. In this context, nanotechnology was suggested as a feasible approach for optimizing the delivery of drugs indicated to prevent and treat viral infections, including COVID-19 [12,13]. Inhaled drug-loaded nanoparticles have been shown to increase treatment efficacy and reduce side-effects [14]. Nanostructured lipid carriers (NLCs) are new pharmaceutical formulations made up of physiological and biocompatible lipids, surfactants, and co-surfactants. NLCs have several critical characteristics that make it a viable drug delivery system: simplicity of manufacture, biocompatibility, scale-up feasibility, nontoxicity, increased drug loading, and stability [15]. The present study was designed to formulate and evaluate HCQ using NLCs (HCQ-NLCs).

A nanoformulation of HCQ was recently reviewed. Several nanoformulations were described, including liposomes, polymeric micelles, polymeric nanoparticles, dendrimers, niosome gel, and titanium dioxide nanoparticles. The efficacies of some of these formulations were tested in a tumor model [16]. Therefore, there is a knowledge gap in the use of HCQ-NLCs in the management of acute lung injury.

## 2. Materials and Methods

### 2.1. Materials

Hydroxychloroquine (sulfate) and sweet almond oil (Sigma-Aldrich Co., St Louis, MO, USA). Compritol^®^ 888 ATO (glyceryl behenate) and Gelucire^®^ 44/14 (GattefosséSaint-Priest, France). L-phosphatidylcholine (soya 95%) (Avanti Polar Lipids, Birmingham, UK). Dexamethasone (8 mg/2 mL injection) (Amrita Pharmaceutical Industries, Alexandria, Egypt). Bleomycin Sulfate (15 units/vial) (Korea United Pharm. Inc). The Enzyme-Linked Immunosorbent Assay (ELISA) kits: tumor necrosis factor-alpha (TNF-a), Interleukin 6 (IL-6), Interleukin 1 beta (IL-1β), and nuclear factor kappa B (NF-κB) (My-bio-source Inc., San Diego, CA, USA). Kits for protein assay (Abcam, Cambridge, UK).

### 2.2. Synthesis of HCQ-NLC

HCQ-NLC formulations were prepared by the hot emulsification–ultrasonication method (Kar, Chakraborty, et al., 2017; Fahmy, Ahmed, et al., 2020). The total lipid content was kept constant at 10% *w*/*v*. Sweet almond oil 100 mg (liquid lipid, LL), glyceryl behenate 900 mg (solid lipid, SL), L-phosphatidylcholine 200 mg (amphiphilic emulsifier, 2% *w*/*v*), and 50 mg of HCQ were mixed and heated to 70 °C with stirring to form a homogenous lipid phase. The aqueous phase was generated by the dissolution of 150 mg of Gelucire^®^ 44/14 (hydrophilic emulsifier, 1.5% *w*/*v*) in distilled water heated to 70 °C, then added dropwise to the melted lipid phase. The obtained dispersion was homogenized (20,000 rpm at 70 °C for 3 min) by an IKA Ultra-Turrax T8 homogenizer (IKA, Wilmington, NC, USA), followed by ultrasonication of the pre-emulsion using a Sonics Vibra Cell VCX750 (Sonics & Materials Inc., Newtown, CT, USA) for 2 min. The HCQ-NLC formulations were stored in a refrigerator at 8 °C for further investigations within 7 days.

### 2.3. Characterization of HCQ-NLC

#### 2.3.1. Particle Size, PDI, and Zeta Potential

Assessment of the particle size, zeta potential, and polydispersity index (PDI) of the prepared HCQ-NLC formulation was carried out by a Malvern size analyzer (Nano ZSP, Malvern Analytical, Malvern, United Kingdom). Samples of the formulation were adequately diluted with the aqueous phase before measurement to obtain an optimum count of 50–200 kilo-counts per second (kcps). Data represent the average of five measurements of all tested parameters.

#### 2.3.2. In Vitro Release Study

The dialysis bag technique was adopted to study the in vitro release of HCQ from its NLC formulations. The specified quantity of HCQ nanoformulations (equivalent to 10 mg of HCQ) was put in the dialysis bags (14 kDa molecular weight cut-off (MWCO)) and then suspended in 500 mL of distilled water (sink condition) at 37 °C while rotating at a speed of 75 rpm (USP Dissolution Tester, apparatus II (Erweka, Germany)). Five-milliliter aliquots were taken from the dissolution medium at specified time points, 0.5, 1, 2, 4, 6, 8, 12, and 24 h. Three samples were tested, and concentrations of HCQ were determined by the high-performance liquid chromatography (HPLC) method. Pure HCQ sulphate showed complete dissolution within 5 min (data not shown).

### 2.4. HPLC Analysis of HCQ

The HCQ concentration was determined by the HPLC method, which was validated in our labs in terms of accuracy, linearity, and precision. Analysis was performed using the Agilent 1200 system with a diode array detector, Zorbax Extend C18 column (4.6 mm × 150 mm, 5 µm), quaternary pump, and auto-sampler (Palo Alto, CA, USA). The system was controlled by ChemStation software (Rev. B.01.03 SR2 (204). Isocratic elution at a 0.6 mL/min flow rate was utilized. The mobile phase was composed of acetonitrile/water acidified with 0.1% formic acid (9:1). The wavelength of the detector was set at 343 nm (peak absorbance of HCQ).

### 2.5. In Vivo Evaluation

#### 2.5.1. Animal and Experimental Design

**Figure 1:**
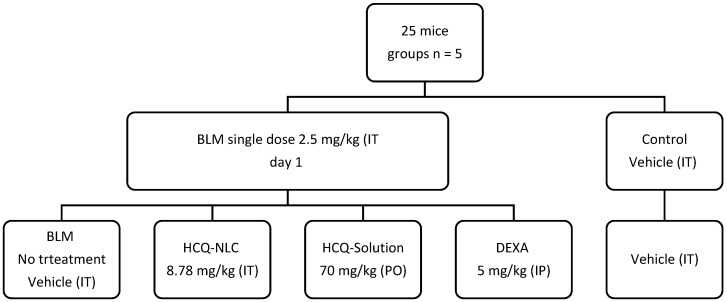
Animal experiment design.

The study protocol was approved by the ethical committee at KAU, Faculty of Pharmacy with approval no “PH-1442-71”. A total number of 25 male, 3-months-old mice, with an average weight of 25 g, were used. All mice received good care complying with ethical standards. Mice were randomly divided into 5 groups, 5 mice in each group, as presented in Figure 1.

Acute lung injury was induced by using a single dose of BLM IT (2.5 mg/kg) [17]. Then, this model was treated for 6 successive days by the following: HCQ-NLC IT (8.78 mg/kg), oral formulations of HCQ suspension (70 mg/kg), or dexamethasone (DEXA) (5 mg/kg) intraperitoneal injection (IP). Three control groups were used (control): animals receiving only vehicle for NLC IT; the 2nd BLM IT only; DEXA (5 mg/kg, IP) was used as a positive control. The dose of HCQ was chosen based on the recommended human dose for COVID-19 [18,19] considering the limited ability of the animal’s lungs to tolerate inhaled fluids and the general assumption of the increased lung bioavailability of drugs given by inhalation [20].

Corticosteroids are known for their inhibition of the inflammatory cascade. The dose of DEXA as a positive control was chosen based on the reported value [21].

At the end of the experiment, the animals were sacrificed and the lungs were collected; the right lung was processed for pro-inflammatory cytokines analysis by ELISA and the left lung was processed for histological investigations.

#### 2.5.2. Preparation of Oral HCQ Solution

The HCQ (Sulphate) solution was prepared daily as follows: 40 mg of the HCQ powder was dissolved in 5 mL of distilled water (8 mg/mL).

### 2.6. Pro-Inflammatory Markers Analysis

Measurements of TNF-α, IL-6, IL-1β, and NF-κB values were assessed by using mouse ELISA kits. The procedure and calibration were performed as guided by the kit manufacturer. All assessed pro-inflammatory markers in lung homogenate were expressed per (μg protein) after measuring the total protein in each sample.

### 2.7. Preparation of Tissue Homogenate

Ice-cold phosphate-buffered solution (PBS) 0.01 M with a pH of 7.4 was used to wash the lung tissue to remove clotted blood. An amount of 100 mg of lung tissue was homogenized in 1 mL of PBS and stored immediately at −20 °C for 1 h. Subsequently, the cell membranes were ruptured with two freeze–thaw cycles; then, centrifugation (5000 rpm, 5 min) of the homogenates was carried out at −8 °C. The supernatant was aspirated and stored at −80 °C. Before any assays, the specimens underwent centrifugation, but no further freeze–thaw cycles [22].

### 2.8. Histopathological Evaluation

Samples from the left lung tissues underwent fixing in 10% formalin solution. The specimens were then dehydrated, cleared, and embedded in paraffin. Consecutive slices of 5 µm were sectioned and then stained with hematoxylin and eosin for the histopathological assessment using an Olympus light BX61 microscope by a histologist [23].

### 2.9. Statistical Analysis

Statistical analysis was performed using the Statistical Package for the Social Sciences software (version 23, SPSS, Chicago, IL, USA). One-way analysis of variance (ANOVA) followed by Tukey’s post hoc test was used for multiple comparisons. The statistically significant difference between the mean values was considered (*p* < 0.05). Values in the text and tables were represented as means ± standard deviation (M ± SD). Graphs were sketched using GraphPad Prism software version 8 (GraphPad^®^ Inc., San Diego, CA, USA).

## 3. Results

### 3.1. Characterization of HCQ-NLC

**Figure 2:**
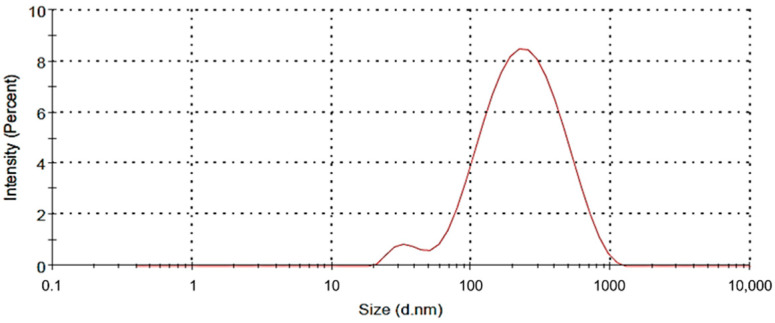
Particle size distribution of HCQ-NLC.

HCQ-NLCs were successfully prepared by the hot emulsification–ultrasonication method using L-phosphatidylcholine as a surfactant. The resultant nanoparticle size, zeta potential, and PDI were 277 ± 10 nm (Figure 2), 2.6 ± 0.2 mV, and 0.293 ± 0.02, respectively. These results were an average of three experiments, each performed in triplicate.

**Figure 3:**
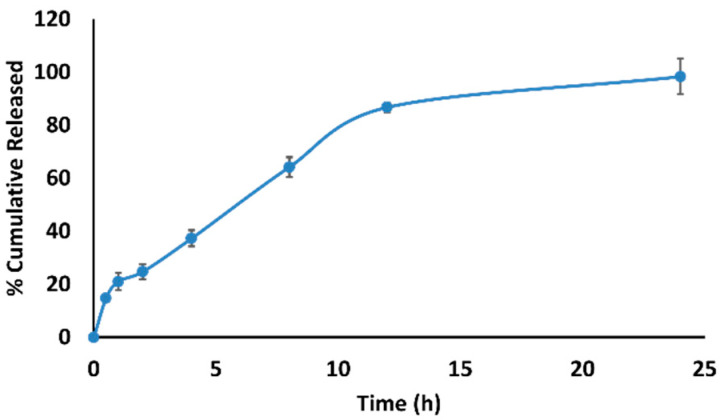
In vitro drug release profile of HCQ-NLC at 37 °C.

To determine the pattern of drug release from the nanoparticle, in vitro drug release studies illustrated about 15% of drug release in 30 min followed by a cumulative release of above 80% after 12 h (Figure 3).

### 3.2. Pro-Inflammatory Markers Analysis

**Figure 4:**
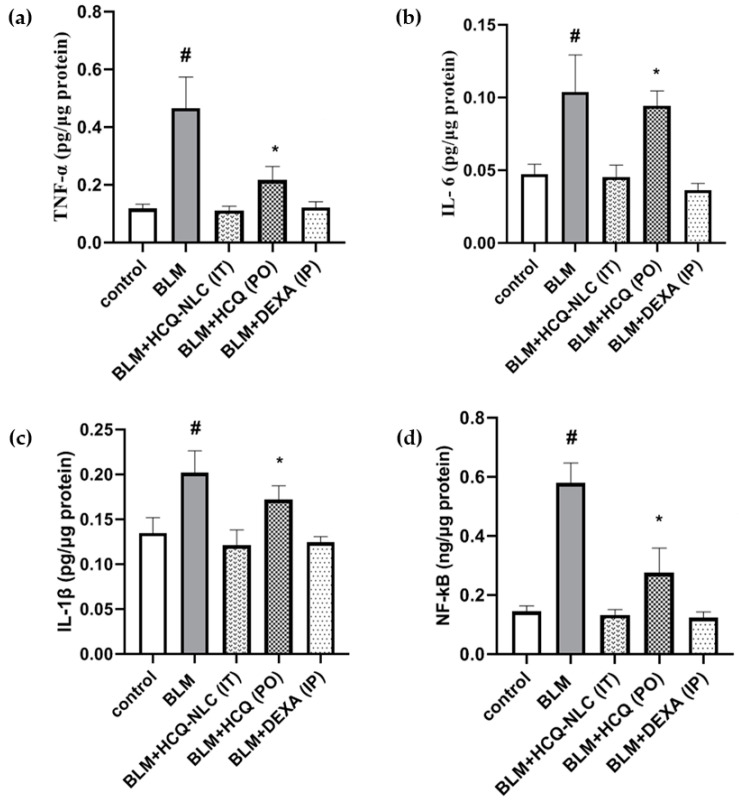
Effect of various HCQ formulations on the concentration of (**a**) TNF-α, (**b**) IL-6, (**c**) IL-1β, and (**d**) NF-κB in lung tissues. Animals were given a single dose of BLM (2.5 mg/kg) followed by, once daily, six doses of either HCQ-NLCs (8.78 mg/kg) intratracheally (IT), HCQ (70 mg/kg) orally (PO), or DEXA (5 mg/kg) intraperitoneally (IP). # Significant (*p* < 0.05) versus all groups. * Significant (*p* < 0.05) versus the control group and HCQ-NLC group.

To mimic the pathological changes associated with complicated COVID-19, a BLM-induced acute lung injury mice model was used. A single dose of 2.5 mg/kg of BLMIT resulted in a significant rise in the concentration of TNF-α, IL-6, IL-1β, and NF-κB in lung tissue homogenate (*p* < 0.05). Treatment with HCQ-NLCs IT at the dose of 8.78 mg/kg significantly attenuated the levels of TNF-α, IL-6, IL-1β, and NF-κB, bringing them back to the levels seen in the negative control group. Although HCQ PO at the dose of 70 mg/kg decreased the levels of the pro-inflammatory markers, the reduction seen with the pulmonary delivery of HCQ-NLCs was significant when compared with the HCQ-PO-treated group (Figure 4).

### 3.3. Histopathological Changes

**Figure 5:**
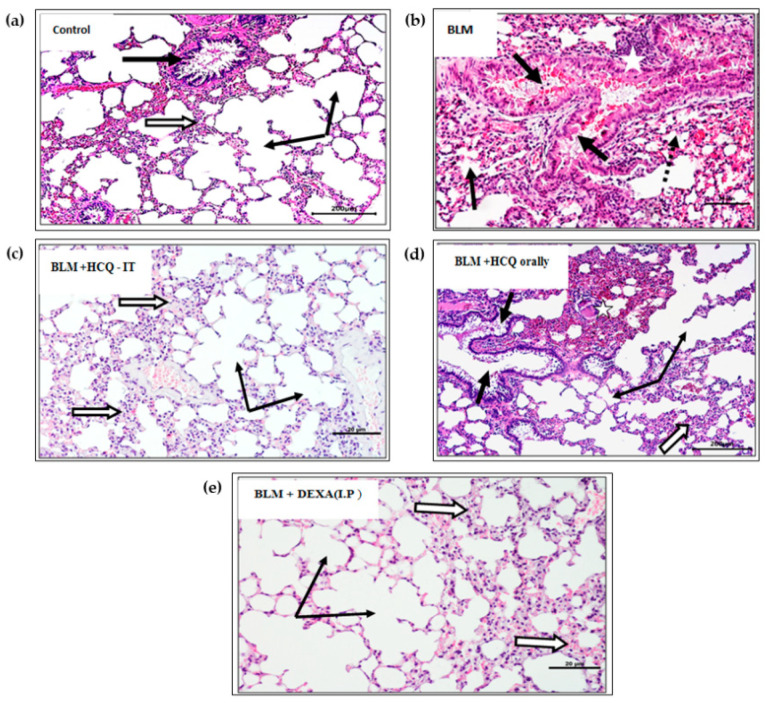
Sections of lung tissues stained by H&E and photographed at X100 (Bar = 200 µm) after administration of HCQ-NLC IT compared with different control groups. (**a**) Control group: The alveoli were free of any inflammatory cells, normally opened (thin black arrows), and separated by thin inter-alveolar septa. A nearby bronchiole showed a normal lining and empty lumen (white arrow). (**b**) BLM: Bronchioles (thick black arrows) contained epithelial lining degeneration with desquamation and hemorrhages that block their lumina (white stars). The alveoli showed marked disorganization and looked collapsed or filled with inflammatory cells. A congested capillary was observed (dotted arrows). (**c**) BLM + HCQ-NLC (IT): Opened alveoli free of any inflammatory deposits were shown. The only focal thickening of the inner-alveolar septa was shown (white arrows). (**d**) BLM + HCQ orally: A moderate improvement was shown, with opened alveoli, but many foci of inflammatory cell aggregates were closing nearby alveoli (white star). Bronchioles contained scanty exudates (black arrow). (**e**) BLM + DEXA (IP): A marked improvement was shown, with thin interalveolar septa and few connective tissue cells (white arrow).

The lung tissues of animals that received the vehicle nanoformulations (Control) showed all normal features of healthy mice. Alveoli showed normally opened lumina and the absence of inflammatory cells, which were separated by thin inter-alveolar septa, containing scanty connective tissue cells in addition to thin-walled noncongested capillaries. The bronchioles showed a normal appearance, intact epithelial lining, and empty lumen (Figure 5). The BLM group (acute lung injury animal model) showed histological changes in bronchioles including epithelial lining degeneration with desquamation and hemorrhages that block their lumina. The alveolar structure showed marked disorganization and looked collapsed or filled with inflammatory cells. The interalveolar septa were thickened and showed massive inflammatory cell infiltration. The congested capillary was occasionally observed. The lung of animals receiving IT HCQ-NLCs looked histologically nearly healthy compared to the nontreated BLM group; it showed opened alveoli with almost normal features, free of any inflammatory deposits. However, few focal thickenings of inner-alveolar septa were observed. The oral administration of HCQ also resulted in moderate or mild improvements of the histological alterations induced by BLM. Most alveoli were open, but many foci still showed inflammatory cell aggregates closing nearby alveoli. Bronchioles contained scanty inflammatory exudates (Figure 5). The lung of mice treated with the IP administration of DEXA showed a marked improvement, where alveoli and their lumina were free of any inflammatory infiltrate. Interalveolar septa looked thin and free of inflammatory cells.

## 4. Discussion

Most available drugs for the management of COVID-19 showed drawbacks. HCQ received extensive debate regarding its efficacy and safety in the management of COVID-19 [24]. The major limitations after oral administration include low cellular uptake in the case of lung acidosis induced by severe COVID-19, and extreme variability in drug levels [10]. Its cardiac toxicity is likely to be exaggerated in COVID-19 and the concomitant use of cardiotoxic drugs such as azithromycin [25]. Optimized lung delivery systems allow drugs to selectively target lung tissues, enhancing their efficacy with minimal systemic toxicity, to ensure a fast effect, and to decrease the variability in drug exposure. These favorable features could effectively optimize the pharmacotherapy of COVID-19 [26,27]. Nanoparticle-based delivery systems were suggested for their utility to optimize the pharmacotherapy of COVID-19 [28]. Therefore, based on the above criteria and the low cost of HCQ, the formulation and evaluation of inhaled NLCs of HCQ is a rational approach to optimize its use in the management of COVID-19.

In this study, the HCQ-NLC was prepared by the hot emulsification–ultrasonication method [29]. Almond oil and Compritol^®^ 888ATO were chosen based on their documented success in the formulation of NLCs. The used lipid substances are proven for their good biodegradability and biocompatibility. Moreover, almond oil is a natural oil that has a good safety profile. These lipids have documented advantages for the formulation of NLCs by several studies [26,30,31]. L-phosphatidyl choline was used as an amphiphilic surfactant to promote NLC stability. Moreover, the combination of lipophilic and hydrophilic surfactants is proven to cause decreased particle sizes in comparison to being used either alone [32]. HCQ-NLCs showed acceptable in vitro characteristics, particle sizes, PDI, zeta potentials, and sustained release patterns.

In the current study, the potential efficacy of the prepared HCQ-NLC was evaluated in BLM-induced acute lung injury, which is the most widely used model for studying the impact of drugs on the management of pulmonary injury [33]. BLM generates a well-established murine model of lung inflammation with pathophysiologic changes including the stimulation of cytokines in lung tissues likely similar to that of COVID-19 [34,35].

We used endotracheal instillation of a single dose of BLM IT (2.5 mg/kg), resulting in the development of acute lung injury within 5 days of administration [17,36]. Excessive cytokine production played a basic role in mediating the pathogenesis of acute lung disorder. These inflammatory cytokines induced by BLM precede the structural lung lesion [37,38]. Excessive NF-κB stimulation plays a fundamental role in the inflammatory response [37,39]. TNF-α plays an important role in leukocytes recruitment and lung injury. It exacerbates inflammation, causes damage, and recruits neutrophils to the lung in acute lung injury [37]. IL-6 is a pro-inflammatory cytokine that is a well-documented mediator of numerous inflammatory pathways in the respiratory tract [40]. IL-1β is another pro-inflammatory cytokine that affects cellular damage. Its activation may exacerbate the inflammatory response and leukocyte recruitment to give rise to epithelial abnormalities within the lung tissue [37]. HCQ has been proven to have anti-inflammatory and immunomodulatory effects in several studies [5]. HCQ has been documented to exert anti-inflammatory activity via down-regulation of pro-inflammatory cytokines [41,42]. Moreover, HCQ has a potential therapeutic role in inflammation related to pulmonary fibrosis [43,44].

In our study, the concentrations of these pro-inflammatory biomarkers (TNF-α, IL-6, IL-1β, and NF-κB) were markedly elevated in lung tissue homogenate of the BLM mice model of acute lung injury. The administration of HCQ nanoformulations by the IT route at a dose of 8.78 mg/kg significantly attenuated the BLM-induced elevation in these pro-inflammatory biomarkers. This effect is superior to that observed after oral administration of HCQ at the higher dose (70 mg/kg). The attenuation effects of HCQ on pro-inflammatory biomarkers were reported in previous studies [42,43,45].

These results highlight the role of the inhalation route in the treatment of pulmonary disease and its utility for targeted drug delivery to lung tissues, the high bioavailability, and the rapid onset of action [26,28,46,47].

In the current study, histological assessments of the lung tissues confirmed the acute lung injury induced by BLM in mice. These BLM-induced histological changes have been reported by several studies [36,37]. All these histological changes were ameliorated after treatment with HCQ-NLCs. The present results of HCQ on lung tissue were similarly reported by several studies [43,44]. The enhanced effect of lung delivery of the HCQ nanoformulation is explained given the enhanced access of the drug to lung tissues. These features of lung delivery are documented in several publications [26,28,46].

## 5. Conclusions

A nanoparticle-based drug delivery system of HCQ was successfully prepared by using the hot emulsification–ultrasonication method. The prepared NLC provided adequate targeting of the drugs to lung tissues. This novel strategy will eventually improve its effectiveness and diminish adverse drug reactions of the drug. Lung delivery could be a promising treatment for acute lung injury regardless of its cause; however, further work is needed to explore the stability of the formulation, its pharmacokinetics, and safety.

## Data Availability

The data presented in this study are available on request from the corresponding author.
